# A high-grade breast mucoepidermoid carcinoma without MAML2 rearrangement: A case report and literature review

**DOI:** 10.1097/MD.0000000000037163

**Published:** 2024-02-23

**Authors:** Liangliang Wang, Dan Cheng, Huaying Wang, Lin Cheng, Xiaorong Zhang

**Affiliations:** a Department of Pathology, Affiliated Hospital of Jiujiang University, Jiujiang, Jiangxi 332000, China; b Department of Neurology, Affiliated Hospital of Jiujiang University, Jiujiang, Jiangxi 332000, China.

**Keywords:** breast, MAML2 protein, mucoepidermoid tumor, prognosis

## Abstract

**Introduction:**

Mucoepidermoid carcinoma (MEC) of the breast is an extremely rare primary breast tumor. Between 1979 and June 2022, only 50 cases were reported. The pathological morphology and biological behavior of breast MEC remain poorly understood.

**Patient concerns:**

A 47-year-old female was presented with a 10-day-old left breast mass detected by physical examination.

**Diagnoses:**

Ultrasonography could not distinguish whether the breast tumor was benign or malignant. After a biopsy of a breast tumor excision specimen, combined with immunohistochemical results, the patient was diagnosed with high-grade mucoepidermoid breast carcinoma.

**Interventions:**

The patient underwent a modified radical mastectomy for her left breast.

**Outcomes:**

The patient was still free from local recurrence or metastases at 1-year follow-up.

**Conclusion:**

A high-grade MEC case without MAML2 rearrangement shows good recovery without complications. The diagnosis was confirmed by histomorphology and immunohistochemical markers. It is sometimes necessary to distinguish it from adenosquamous, adenoid cystic, or mucinous carcinoma. The primary treatment is surgical resection, and the prognosis is closely related to the pathological grade.

## 1. Introduction

Mucoepidermoid carcinoma (MEC) is an extremely rare primary breast tumor, with an estimated incidence of only 0.2% to 0.3% of all primary breast tumors.^[1]^ It was first reported in 1979,^[2]^ and only about 50 cases have been reported in the literature. The pathological morphology and biological behavior remain poorly understood. Recently, researchers have paid more attention to breast MEC, particularly its imaging features and pathology.^[3–5]^ Bui et al^[6]^ first described MEC in a 50-year-old woman with adenomyoepithelioma this year. According to the literature review, its morphology and biological behavior are similar to mucoepidermoid carcinoma of the salivary gland. In the diagnosis process, it is sometimes necessary to distinguish it from adenosquamous carcinoma, adenoid cystic carcinoma, and mucoepidermoid carcinoma. Due to the small number of reports, the clinical behavior and prognosis of this tumor remain unclear.

## 2. Case report

**Figure 1.**
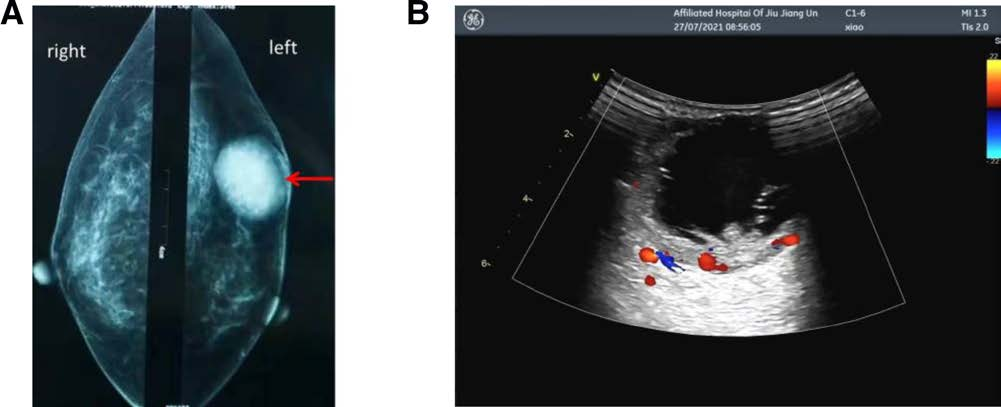
The mammography and Doppler imaging of the left breast of the patient. (A) Mammography shows 45 × 37 mm clear boundaries and hyperdense mass (arrowheads) in the left breast; microcalcifications are undetected in the mass. (B) Internal vascularity identified in the mass on color Doppler imaging.

**Figure 2.**
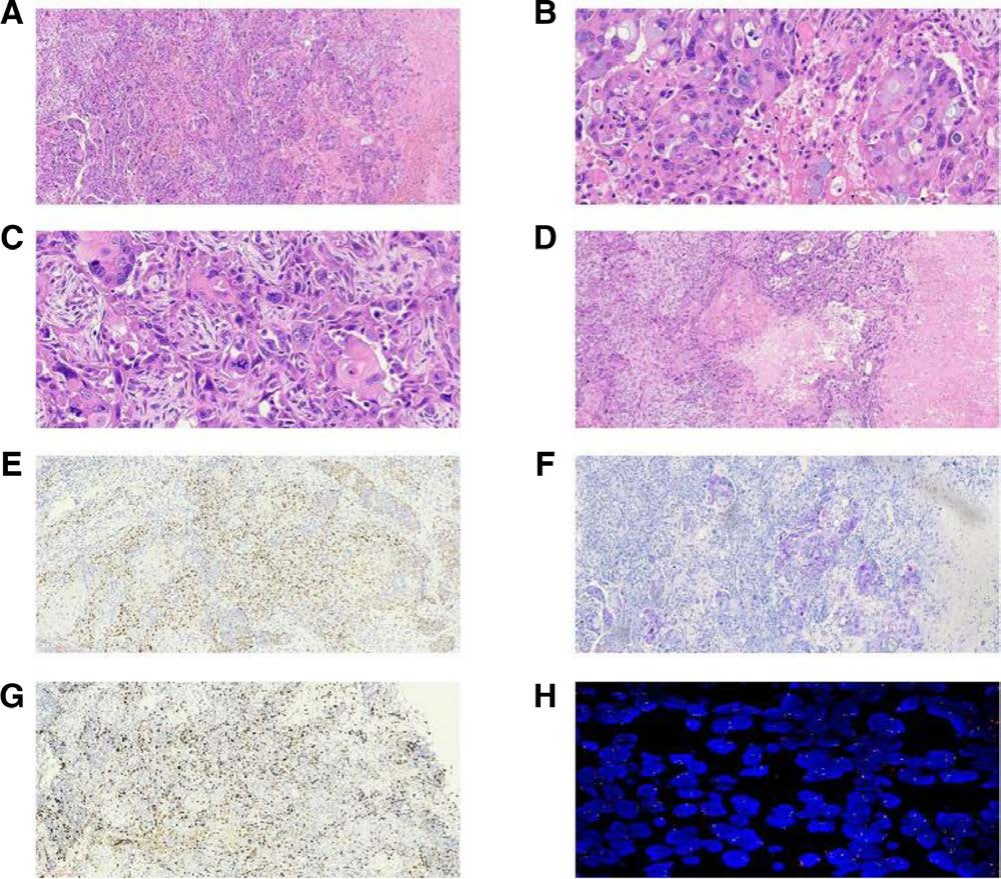
HE, immunohistochemistry, and FISH of breast mucoepidermoid carcinoma. (A) The lesion is composed of intermediate cells, squamous neoplastic cells, and mucinous in varying proportions (HE, original magnification 100×). (B) The mucinous cells are characterized by many mucinous (HE, original magnification 400×). (C) The squamous neoplastic cells and mitotic figures (arrowheads) (HE, original magnification 400×). (D) Necrosis (HE, original magnification 100×). (E) p63 positive (immunohistochemistry, original magnification 100×). (F) PAS positive (special PAS staining, original magnification 100×). (G) A ki-67 stain showed a high proliferation of about 30% (immunohistochemistry, original magnification 100×). (H) A fusion of orange and green signals > 90% of the lesional cells (FISH). FISH = fluorescence in situ hybridization, HE = hematoxylin-eosin.

A 47-year-old female was presented with a 10-day-old left breast mass detected by physical examination. CT examination revealed a rounded low-density nodular shadow measuring 45 × 37 mm in the left breast, without associated microcalcifications and architectural distortion. Ultrasonography revealed a cystic, solid echo area of 45 × 37 mm at 2 o’clock in the left breast with clear boundaries, separated light bands, and many small echo spots in the mass. Mammography displayed a hyperdense mass with clear boundaries in the left breast with no microcalcifications (Fig. 1A). Internal vascularity was identified by color Doppler imaging (Fig. 1B). To completely excise the mass, a lumpectomy plus sentinel lymph node biopsy was performed without lymph node metastasis. The gross specimen exhibited a 45 × 37 × 20 mm gray and white solid nodule with a clear boundary in the upper quadrant of the left breast at 2 o’clock. Under the microscope, the tumor tissue was lobulated with many cysts of different sizes. Some mucous can be seen in the cavities. The lesion was composed of varying proportions of intermediate, squamous neoplastic, and mucinous cells (Fig. 2A). The intermediate cells were characterized by eosinophilic cytoplasm and a medium nuclear-to-cytoplasmic ratio. The mucinous cells were characterized by many mucinous in the cytoplasm with PAS positive (Fig. 2B). The squamous neoplastic cells were heteromorphic, and individual cell keratinization and mitotic figures were common (Fig. 2C). Necrosis (Fig. 2D) and neural invasion were observed in the lesion. We also observed a prominent lymphoplasmacytic infiltrate around the lesion, with no evidence of lymph node metastasis.

Squamous and intermediate cells were positive for p63 (Fig. 2E) and CK5/6. The mucinous cells were positive for PAS (Fig. 2F). AR (20%+) and estrogen receptor (1%+) were expressed at low levels in the lesion. The tumor cells with progesterone receptor, HER2, CD56, CgA, and Syn staining were negative. A ki-67 stain revealed a high proliferation of about 30% (Fig. 2G). Fluorescence in situ hybridization revealed a fusion of orange and green signals in more than 90% of lesion cells, indicating no rearrangement of MAML2 (Fig. 2H). The pathological diagnosis was a high-grade breast MEC. The patient was subsequently followed-up for a year without local recurrence or distant metastasis.

## 3. Discussion

Because the breast and major salivary glands are derived from the embryonal ectoderm, the salivary gland and breast tumors are sporadic and similar. They have the same basic tubuloalveolar structures, probably explaining the similar morphological features of tumors.^[7,8]^ Therefore, breast malignant salivary gland tumors, like those in the major salivary glands, can be divided into two groups according to the absence or presence of myoepithelial differentiation.^[9]^ Tumors without myoepithelial differentiation include MEC, oncocytoma, and acinar cell carcinoma, while those with myoepithelial differentiation include adenosquamous carcinoma, adenoid cystic carcinoma, and pleomorphic adenoma. MEC is one of the rare breast tumors, accounting for only 0.2% to 0.3% of all breast cancers.^[10]^ Herein, we summarized all breast MEC cases reported between 1979 and 2021 (Table 1) and found that only 49 cases had been reported in the literature. All patients were female in these cases, ranging from 27 to 86 years (median, 56.14 ± 11.1 years). A breast MEC usually ranges from 0.5 to 10 cm, with a clear boundary; however, the border may be unclear if the mass is very large. The clinical presentation was painless or painful solid-cystic mass with a clear margin. Mammography always demonstrated circumscribed dense or solid-cystic masses without associated microcalcifications. Without lymph node metastasis, the surgery was always mastectomy or lumpectomy plus sentinel lymph node biopsy.

**Table 1:** A summary of 48 cases of MEC of the breast reported in the English language literature from 1979 to 2022.

No.	Author [ref.]	Year	Age (yr)	Size (cm)	Surgical treatment	Grade	LM	DM	Genetic test	Follow-up (mo)	Status
1	Present study	2022	42	5.0	MRM	HG	0/4	No	No MAML2 (FISH)	12	Alive
2	Chen et al^[5]^	2022	38	NA	Lump	LG	NA	No	NA	6	Alive
3	Bak et al^[1]^	2022	47	3.2	Lump + LND	IG	0/NA	No	NA	37	Alive
4	Bai et al^[3]^	2022	63	NA	SM + SLND	IG	0/NA	No	NA	6	Alive
5	Ye et al^[4]^	2020	42	2.6	MRM	LG	0/NA	No	NA	12	Alive
6	Yan et al^[9]^	2020	60	1.9	Lump	LG	NA	No	MAML2 (FISH)	60	Alive
7	Brunac et al^[11]^	2019	51	2.5	Lump	LG	0/NA	No	No MAML2 (FISH)	12	Alive
8	Bean et al^[12]^	2019	53	0.9	MR	LG	0/NA	No	MAML2 (FISH)	16	Alive
9		2019	49	5	MRM	IG	1/NA	No	NA	12	Alive
10	Burghel et al^[13]^	2018	73	NA	Lump + LND	LG	0/2	No	APC mutation (Sanger)	NA	NA
11	Sherwell et al^[14]^	2017	86	7	MRM	LG	0/NA	No	NA	3	Alive
12	Cheng et al^[7]^	2017	39	1.5	MRM	LG	3/18	No	NA	156	Alive
13			49	1.5	MRM	LG	0/17	No	NA	41	Alive
14			66	1.3	SM + SLND	LG	0/6	No	NA	9	Alive
15			61	3.0	SM + SLND	LG	0/3	No	NA	4	Alive
16	Fujino et al^[15]^	2016	71	2.0	SM + SLND	IG	0/NA	No	No MAML2 (PCR)	NA	NA
17	Palermo et al^[16]^	2013	80	4	Lump	HG	0/NA	No	NA	NA	NA
18	Turk et al^[17]^	2013	40	4	MRM	NA	1/24	No	NA	1/24	Alive
19	Basbug et al^[18]^	2011	69	10	MRM	HG	0/12	No	NA	0/12	Alive
20	Camelo-Piragua et al^[10]^	2009	49	4.0	MRM	IG	1/3	No	11q21 partial loss (FISH)	12	Alive
21	Hornychova et al^[8]^	2007	63	1.8	SM + LND	HG	0/17	No	NA	18	Alive
22			30	8.0	MRM	LG	0/NA	No	NA	60	Alive
23	Horii et al^[19]^	2006	54	2.5	MRM	LG	0/NA	No	NA	36	Alive
24	Gomez-Aracil et al^[20]^	2006	69	6.0	MRM	HG	24/28	No	NA	54	Alive
25	Terzi et al^[21]^	2004	79	8.0	MRM	HG	4/14	NA	NA	NA	NA
26	Di Tommaso et al^[22]^	2004	80	0.5	Lump	LG	NA	No	NA	5	Alive
27			29	0.8	Lump	LG	NA	No	NA	90	Alive
28			54	1.5	Q + LND	LG	0/NA	No	NA	13	Alive
29			36	1.1	Q + LND	HG	0/NA	No	NA	18	Alive
30			55	0.6	Q + LND	IG	0/NA	No	NA	3	Alive
31	Tjalma et al^[23]^	2002	58	3.5	RM	HG	1/17	Yes	NA	156	Alive
32	Berry et al^[24]^	1998	51	3.5	RM	HG	0/NA	No	NA	NA	NA
33	Markopoulos et al^[25]^	1998	40	2.0	MRM	HG	0/NA	No	NA	60	Alive
34	Chang et al^[26]^	1998	54	4.5	MRM	HG	0/9	No	NA	48	Alive
35	Luchtrath et al^[27]^	1989	60	5.0	RM	HG	12/18	Yes	NA	30	DOD
36	Pettinato et al^[28]^	1989	72	7.0	MRM	HG	16/19	Yes	NA	10	DOD
37	Hanna et al^[29]^	1985	51	2.0	MRM	NA	0/NA	No	NA	8	Alive
38			31	NA	MRM	NA	2/18	No	NA	14	Alive
39	Hastrup et al^[30]^	1985	59	1,0	RM	HG	0/4	Yes	NA	25	DOD
40	Ratanarapee et al^[31]^	1983	27	NA	NA	HG	6/15	Yes	NA	14	DOD
41	Fisher^[32]^et al	1983	65	2	Lump	LG	NA	No	NA	60	Alive
42			71	NA	NA	LG	0/19	No	NA	48	Alive
43			57	2.5	MRM	LG	0/11	No	NA	120	Alive
44			49	3.7	RM	LG	0/13	No	NA	108	Alive
45			60	4.0	SM	LG	NA	No	NA	48	DOR
46	Kovi et al^[33]^	1981	46	11	MRM	HG	17/19	NA	NA	NA	NA
47	Patchefsky et al^[2]^	1979	66	1.3	RM	LG	0/20	No	NA	94	DOR
48			70	5.0	Q	LG	NA	No	NA	10	Alive

DM = distant metastasis, DOD = died of disease, DOR = died of other reason, FISH = fluorescence in situ hybridization, HG = high grade, IG = intermediate grade, LG = low grade, LM = lymphatic metastasis, LND = lymph node dissection, Lump = lumpectomy, MEC = mucoepidermoid carcinoma, MRM = modified radical mastectomy, NA = unavailable, PCR = polymerase chain reaction, Q = quadrantectomy, RM = radical mastectomy, SLND = sentinel lymph node biopsy, SM = simple mastectomy.

Histologically, MEC consists of solid nests or cystic spaces of diverse tumor cell types, including basaloid, intermediate, epidermoid, and mucinous cells. Basaloid cells are usually small, round basophils located at the base or periphery of the nest or cyst. Intermediate cells are slightly larger than basaloid cells, with abundant eosinophilic cytoplasm, and are P63, CK5/6 positive. Epidermoid cells are common in squamous differentiation areas and are P63, CK5/6 positive. The mucinous cells are characterized by many mucinous in the cytoplasm and are PAS positive. However, in high-grade cases, mucinous cells can be absent.

Interestingly, almost all MEC were negative for estrogen receptor, progesterone receptor, and HER2 proteins. Histological classification is determined according to the ratio of cystic components, the presence of neural invasion and necrosis, the mitotic ratio, nuclear grade, border/invasive front, lymphovascular, and bone invasion (Table 2).^[16,34]^ Given their significantly overlapping histomorphologic characteristics, the differential diagnosis of MEC broadly includes intraductal papilloma, clear cell hidradenoma, and adenomyoepithelioma.

**Table 2:** Parameters for histological classification of MEC (Brandwein^[33]^).

Parameter	Point value
Intracystic component < 25%	2
Neural invasion present	2
Necrosis present	3
Mitoses > 5/10 HPF	3
Anaplasia	2
Border/invasive front	2 (small nests and islands)
Lymphovascular invasion	3
Bony invasion	3
Grade	Grade total point score
Low grade	0–4
Intermediate grade	5–6
High grade	>7

HPF = high-power field, MEC = mucoepidermoid carcinoma.

In our literature review, 29 out of 43 (67.5%) cases were low- or intermediate-grade (LG = 23, IG = 6), 17 (34.6%) cases were high grade, and 3 (6.9%) cases grading was unclear. Most breast MECs are low- or intermediate-grade and generally have an indolent behavior and a very optimistic prognosis. The 5-year cancer-specific survival rates of MEC in the parotid gland have been reported as 77% to 97%, which is related to the pathological grade.^[35]^ In comparison, high-grade patients have a poor prognosis with a 5 out of 17 (29.4%) mortality rate after developing distant metastasis 7 to 30 months after diagnosis. The low- or intermediate-grade patients treated with lumpectomy or simple mastectomy may have a very optimistic prognosis. While for high-grade patients with or without distant metastasis, adjuvant radiation therapy may be required. The prognosis was dependent on clinical stage or pathological grade.^[35]^

In summary, breast MEC is a rare condition. Here, we report a high-grade case of breast MEC without MAML2 rearrangement. The MAML2 rearrangement appears in almost all the MEC of salivary; however, it is often lost in MEC of the breast, especially in high-grade MEC.^[36]^ The case pathology reveals a predominant cystic structure with few mucinous cells, necrosis, neural invasion, and a high proliferative index. Therefore, these findings are consistent with a high-grade breast MEC. The patient’s 1-year follow-up shows no local recurrence or distant metastasis. According to our literature review, the clinical presentation was painless or painful solid-cystic mass with clear margins. Moreover, low- and intermediate-grade cases are indolent or relatively benign, while high-grade cases can be fatal, with a mortality rate of 29.4% after developing distant metastasis.

## Acknowledgments

We thank Home for Researchers editorial team (www.home-for-researchers.com) for language editing service.

## Author contributions

**Conceptualization:** Liangliang Wang.

**Supervision:** Xiaorong Zhang.

**Writing – original draft:** Liangliang Wang.

**Writing – review & editing:** Dan Cheng, Huaying Wang, Lin Cheng.
